# Abnormal functional connectivity in psoriasis patients with depression is associated with their clinical symptoms

**DOI:** 10.3389/fnins.2022.1026610

**Published:** 2022-10-13

**Authors:** Xiaoxu Wang, Ni Liu, Lingjun Wu, Yanan Zhang, Guangzhong Zhang

**Affiliations:** ^1^Dermatological Department, Beijing Hospital of Traditional Chinese Medicine, Capital Medical University, Beijing, China; ^2^Graduate School, Capital Medical University, Beijing, China; ^3^Department of Radiology, Beijing Hospital of Traditional Chinese Medicine, Capital Medical University, Beijing, China; ^4^Department of Pediatric, Beijing Hospital of Traditional Chinese Medicine, Beijing, China

**Keywords:** psoriasis, depression, fractional amplitude of low-frequency fluctuation (fALFF), functional connectivity, resting-state fMRI

## Abstract

Psoriasis is a chronic, autoimmune disorder that is related to mental health disorders such as depression. However, few studies have focused on the features of brain activity in psoriasis patients with depression (PPD) and the association between brain activity and disease severity. A total of 29 PPD and 24 healthy controls were involved in this study, and all participants underwent resting-state functional magnetic resonance imaging (fMRI) scanning. The psoriasis area and severity index (PASI) and the self-rating depression scale (SDS) were used to measure clinical symptoms. Compared with HCs, PPD patients showed increased fractional amplitude of low-frequency fluctuation (fALFF) in the Frontal_Mid_L and increased functional connectivity (FC) between the hypothalamus-R and the Cingulum_Mid_R. Correlation analysis suggested a positive correlation between PASI and SDS scores in PPD, while the fALFF and FC values were negatively correlated with their SDS and PASI scores. These brain regions may be associated with the development of depressive symptoms and disease severity in psoriasis patients.

## Introduction

Psoriasis is a common, chronic, autoimmune disease of the skin that is estimated to affect 2% of the worldwide population (Rendon and Schäkel, 2019). And prevalence of psoriasis varies in different ethnic groups. For example, the prevalence in Europe is 1.3–11.4%, Japan is about 0.3–0.4% (Ogawa and Okada, 2020). It is characterized by sharply demarcated, erythematous, pruritic plaques covered in silvery scales and is known to have a significant influence on the quality of life of patients (Kaufman and Alexis, 2018). According to studies, psoriasis patients can easily experience stigmatization and avoid social interaction due to their physical appearance (Dowlatshahi et al., 2014; Jankowiak et al., 2020; Sagaltici, 2020). Thus, psoriasis is also classified as a type of psychosomatic disease that is highly related to psychological comorbidities, such as depression (Tribó et al., 2019; Hölsken et al., 2021). One study investigating 401,703 psoriasis patients showed that 28% of them had depressive symptoms (Dowlatshahi et al., 2014). Another study evaluated 10,932 patients and showed that 46% of psoriasis patients thought the development of their disease was related to stress reactivity, and 54% of patients recalled previous stressful events (Snast et al., 2018). Other studies have shown that patients with depression have an increased risk of psoriasis (Koo et al., 2017; Chen et al., 2021). Furthermore, a decrease in psoriasis severity is related to a decrease in depressive symptoms and vice versa (Polenghi et al., 1994; Fortune et al., 2002; Mease et al., 2010; Menter et al., 2010; Redighieri et al., 2011; Fordham et al., 2015). Although many studies have found a correlation between psoriasis and depression, the underlying pathogenesis of this association is still unknown.

The brain-skin axis is emerging as a useful concept to describe correlations between brain activity and inflammatory skin disease, which may help to improve our understanding of the association between psoriasis and depression (Wang et al., 2021). The hypothalamic–pituitary–adrenal (HPA) axis is a key element of the brain-skin axis, which is the main coordinator of the body’s response to acute or chronic stress (Wang et al., 2021). HPA axis activation in depression could lead to the release of several endocrine hormones, including corticotropin-releasing hormone (CRH), adrenocorticotropic hormone (ACTH), and glucocorticoid (GC) (Slominski et al., 2007). Many studies have shown that the release of these hormones by the HPA axis could aggravate psoriasis and depression progression. For example, some studies found that CRH could contribute to the secretion of several proinflammatory cytokines and the expression of keratinocytes in patients with psoriasis (Vasiadi et al., 2012; Connor et al., 2015). Other studies have indicated that a disorder of the HPA axis is found in 35–65% of patients with depression, and the core feature of these disorders is the hypersecretion of GC (Nemeroff and Evans, 1984; Holsboer, 2000; Swaab et al., 2005; Vreeburg et al., 2009). In addition, the skin has a fully functional peripheral equivalent of the HPA axis. As a skin stressor, psoriasis can stimulate the elements of the skin analog of the HPA axis to contribute to the release of endocrine hormones (Wang et al., 2021). These skin-produced endocrine hormones can affect the HPA axis in the brain, which achieves communication between the brain and skin. Collectively, the above studies suggest that abnormal brain activity in depression could interact with psoriasis through the brain-skin axis. However, few studies have focused on the relationship between abnormal brain activity and the severity of psoriasis.

Currently, resting-state functional magnetic resonance imaging (rs-fMRI) is widely used in the study of brain function abnormalities in humans and is a useful method to explore brain function abnormalities in psoriasis patients with depression (PPD) (Nathan and Bakker, 2021). Many studies have reported brain function abnormalities in depression during the resting state (Greicius et al., 2007; Sheline et al., 2010). For example, Lui et al. (2011) showed that higher functional connectivity between the prefrontal cortex and bilateral thalamus was found in depressive patients. Gao et al. (2021) found that patients with depression have a higher fractional amplitude of low-frequency fluctuations (fALFF) in the right precuneus, left mid cingulum and left superior frontal gyrus than healthy controls. However, few studies have reported abnormal brain activity in psoriasis patients with depression. Thus, it is necessary to investigate the abnormal brain activity of PPD, which could contribute to our understanding of the potential association between psoriasis and depression.

A frequently used method of rs-fMRI is amplitude of low-frequency fluctuation (ALFF), which is thought to reflect changes in spontaneous brain activity and has been applied in many studies of depression diseases (Wang et al., 2022; Zhang et al., 2022). Moreover, fALFF is an analysis method for ALFF standardization, which calculates the ratio of low-frequency amplitude to whole-brain frequency and has been shown to be less susceptible to physiological noise (Zou et al., 2008). After considering the pros and cons of these analysis methods, we decided to investigate local spontaneous neural activity by using fALFF. Functional connectivity (FC) is another commonly used method of rs-fMRI that reflects spatially distinct brain regions’ temporal coincidence (Fornito and Bullmore, 2010). In this study, we used the fALFF and FC methods to investigate brain activity in PPD. Furthermore, the hypothalamus is the first site for signal reception and processing of the HPA axis (Wang et al., 2021). Thus, we used a seed-based correlation method to explore the FC between hypothalamus and whole brain voxels. The clinical symptoms were measured by the psoriasis area and severity index (PASI) and self-rating depression scale (SDS). We hypothesized that PPD will result in abnormal brain activity. We also predicted that the abnormal brain activity and connectivity in PPD would be related to their clinical symptoms.

## Materials and methods

### Subjects

The study was approved by the Medical Research Ethics Committee of our hospital and conformed to the Declaration of Helsinki. The full date of first registration was 05/02/2021, and the registration number was ChiCTR2100043142. All patients and healthy control subjects provided written consent before this study.

Twenty-nine PPD (15 males and 14 females; median age 32 years, interquartile range 12.02 years, age range: 22–54 years) and 24 HCs (10 males and 14 females; median age 32.0 years, interquartile range 13.75 years; age range 22–55 years) participated in this study. All subjects were right-handed, native Chinese speakers. All subjects were recruited from the outpatient department of Dermatology at Beijing Hospital of Traditional Chinese Medicine (BHTCM), China, and were diagnosed by at least two senior dermatologists. Clinical and demographic information are presented in Table 1. All participants completed SDS tests before MRI examinations. Depression was defined by an SDS score ≥ 50 (Zung, 1973). The inclusion criteria for psoriasis patients with depression were (1) age between 18 and 45 years, (2) mild itching, (3) not taking psychotropic medication, (4) no contraindications to fMRI scanning, (5) no history of severe or enduring mental or neurological illness, (6) Self-Rating Anxiety Scale (SAS) < 50 (Zung, 1973) and (7) not receiving probiotics, antibiotics, immunosuppression, or glucocorticoids within 6 months prior to the recruitment date.

**TABLE 1:** Demographic and clinical characteristics of participants.

Measure	PPD (*n* = 29)	HC (*n* = 24)	*P*-value
Gender (male/female)	15/14	10/14	0.470
Age (years)	32 (11)	32 (13.75)	0.795
Educational level (years)	15 (3)	16 (0)	0.867
SDS	55 (11.875)	41.875 (10.625)	< 0.001
SAS	43.75 (10)	41.875 (10.9375)	0.365
PASI	10.3 (8.4)		

SAS, self-rating anxiety scale; HC, healthy controls; PPD, psoriasis patients with depression; SDS, self-rating depression scale; PASI, psoriasis area and severity index.

### Clinical assessment

In this study, psychological state was measured by the SDS and SAS (Yu et al., 2019). All tests were performed within 1 h before each MRI examination.

The PASI was used as a tool to predict the severity of psoriasis. PASI was calculated based on the physician’s assessment of the extent (percentage) and severity (none, mild, moderate, severe, and very severe) of redness, thickness, and scaling locally, such as on the head, trunk, arms, and legs.

### Magnetic resonance imaging data acquisition

Magnetic resonance (MR) imaging was performed by a 3.0 T Siemens Skyra (Germany) in BHTCM. A 20-channel head coil was used, along with foam padding to reduce head motion and scanner noise. All subjects were instructed to be still, keep their eyes open, and stay awake during the MRI scan. First, T2-weighted images were collected to exclude intracranial organic lesions. Then, high-resolution three-dimensional T1-weighted images and functional images were collected with the parameters shown in Table 2. All subjects reported that they did not fall asleep during the scanning period.

**TABLE 2:** MRI scan parameter information.

	T1	EPI	T2
TE (ms)	2.25	30.00	94
TR (ms)	2000	2000	4000
FA (°)	8	90	150
MZ	256 × 256	64 × 64	330 × 330
FOV (mm^2^)	256 × 256	192 × 192	220 × 220
VZ (mm^3^)	1.0 × 1.0 × 1.0	3.0 × 3.0 × 3.0	0.7 × 0.7 × 5
Slices	192	35	20

### Preprocessing of resting-state functional magnetic resonance imaging data

Data preprocessing was performed by using the Resting-State fMRI Data Analysis Toolkit (REST v1.2) in MATLAB R2013b (Chao-Gan and Yu-Feng, 2010). The standard preprocessing steps for the seed-based functional connectivity and independent component analysis were as follows: (1) Removal of the first 5 volumes to allow the participants to adapt to the scanning noise, (2) slice timing, (3) realignment (all head movements exceeding 2 mm were excluded), (4) spatial normalization to the Montreal Neurological Institute coordinate space with 3 mm × 3 mm × 3 mm, (5) spatial smoothing with a 6 × 6 × 6 full-width at half maximum kernel, (6) linear detrending, and (7) regression of the cerebrospinal fluid signal, white matter signal, and six head motions. In addition, for seed-based FC analysis, the data preprocessing should include bandpass filtering (0.01–0.08 Hz).

### Fractional amplitude of low-frequency fluctuation analysis

The timeseries of each voxel were converted into a frequency domain to obtain the power spectrum using a fast Fourier transform (Davis et al., 1989). Z-standardized fALFF (zfALFF) maps of each subject were calculated for the following statistical analysis (Zou et al., 2008).

### Seed-based functional connectivity

Seed-based FC analysis was used to explore brain activity. The hypothalamus was identified in each hemisphere. According to previous studies (Kaufmann et al., 2006; Baroncini et al., 2012; Contreras-Rodríguez et al., 2017), the regions of interest (ROIs) were built as 2-mm radius spheres around the following coordinates: *x* = ± 6, *y* = –10, *z* = –10. For each person, the mean time course within this ROI was calculated as the reference time course (Salvador et al., 2005). Then, a seed-based correlation analysis was performed in a voxelwise manner with the averaged time courses of the whole brain. Individual r-maps were normalized to Z-maps using Fisher’s Z transformation (Liu et al., 2007). Finally, all Fisher’s Z-maps were entered into a two-sample *t*-test to detect the regions showing different FC with the bilateral thalamus between the two groups.

### Statistical analysis

Statistical analysis was performed using IBM SPSS Statistics version 23.0 (SPSS 23.0). The demographic data of the two groups were analyzed by using independent-sample *t*-tests and χ^2^ tests. The comparison of the clinical symptom scores (SDS, PASI) between two groups was analyzed by using a two-sample *t*-test. A *p*-value of 0.05 was considered statistically significant (two-tailed).

Two-sample *t*-tests were performed to determine zfALFF or FC differences between two groups by using statistical parametric mapping 12 (SPM12). Sex, age and years of education were included as regressors of no interest. The results were considered significant at *p* < 0.05 cluster-level familywise error (FWE)-corrected for multiple comparisons after an initial cluster-forming threshold of *p* < 0.001.

Finally, correlation analysis was performed to determine the relationship between zfALFF values or the strength of the FC of significant clusters that survived the two-sample *t*-tests and clinical symptom scores.

## Results

### Clinical and demographic features

The psoriasis patients in the depression and HC groups did not differ significantly in gender (*p* = 0.470), age (*p* = 0.795), or educational level (*p* = 0.867). Details of the demographic data and corresponding tests are presented in Table 1. As shown in Table 1, the SDS scores in psoriasis patients with depression were higher than those in the HC groups. SAS scores did not differ between the two groups.

### Z-standardized fALFF analysis results

**FIGURE 1:**
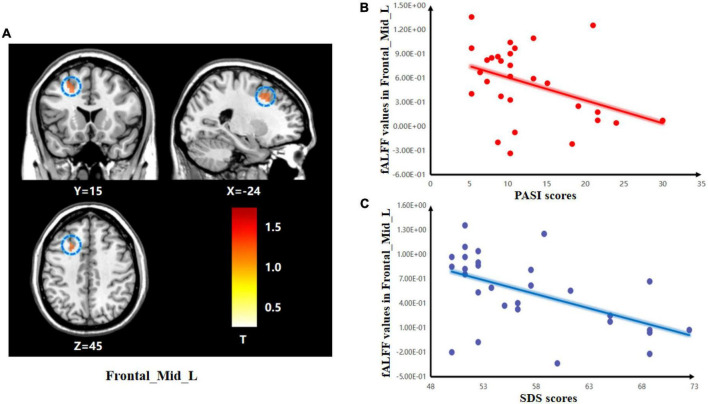
Group differences in the relationships between brain activity and clinical symptoms. **(A)** The two-sample *t*-test showing significant differences in z-standardized fractional amplitude of low-frequency fluctuations (zfALFF) between the two groups. The color bar indicates *T*-values. Correlations between abnormal fractional amplitude of low-frequency fluctuation (fALFF) and PASI and SDS scores. **(B)** Negative correlation between the fALFF values in the Frontal_Mid_L and the PASI scores; **(C)** Negative correlation between the fALFF values in the Frontal_Mid_L and the SDS scores.

The two-sample *t*-test was used to test significant differences in fALFF values between psoriasis patients with depression and healthy controls. Patients showed significantly higher fALFF in the Frontal_Mid_L than healthy controls (Figure 1). Detailed results of the Frontal_Mid_L two-sample *t*-test are presented in Table 3.

**TABLE 3:** Brain region with a significant difference in zfALFF between two groups.

Region	Cluster size	Peak intensity	x	y	z
Frontal_Mid_L	34	5.8676	–24	15	45

### Functional connectivity analysis results

**FIGURE 2:**
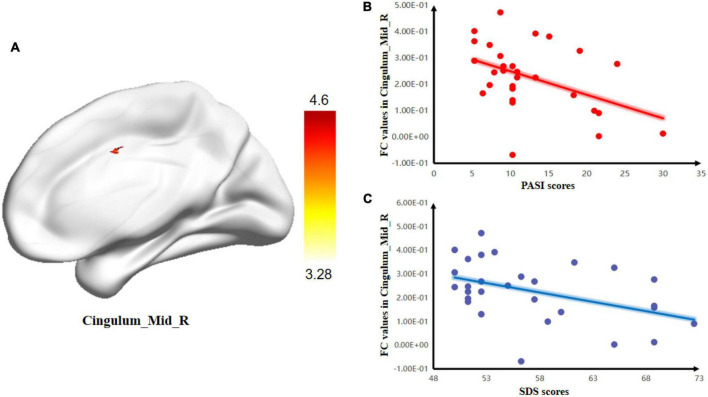
Group differences in the relationships between brain activity and clinical symptoms. **(A)** Comparison between psoriasis patients with depression and healthy controls (HCs) for mean resting-state connectivity between the hypothalamus-R and Cingulum_Mid_R. The color bars indicate *T*-values. Correlations between FC values and PASI and SDS scores. **(B)** Negative correlation between the mean resting-state connectivity values and PASI scores; **(C)** Negative correlation between FC values and SDS scores.

The two-sample *t*-test of the hypothalamus seed-to-voxel FC analysis revealed that, compared with the HC group, psoriasis patients with depression showed a higher positive FC between the hypothalamus-R and the Cingulum_Mid_R (Figure 2 and Table 4).

**TABLE 4:** Brain area with a significant difference in hypothalamus FC between the two groups.

Region	Cluster size	Peak intensity	x	y	z
Cingulum_Mid_R	53	4.5999	3	3	39

Because there was a significant difference in zfALFF in the Frontal_Mid_L between the two groups, we further examined whether the FC of the Frontal_Mid_L with the rest of the brain differed between the two groups. However, there were no group differences for the Frontal_Mid_L seed-to-voxel FC.

### Clinical-magnetic resonance imaging correlations

**FIGURE 3:**
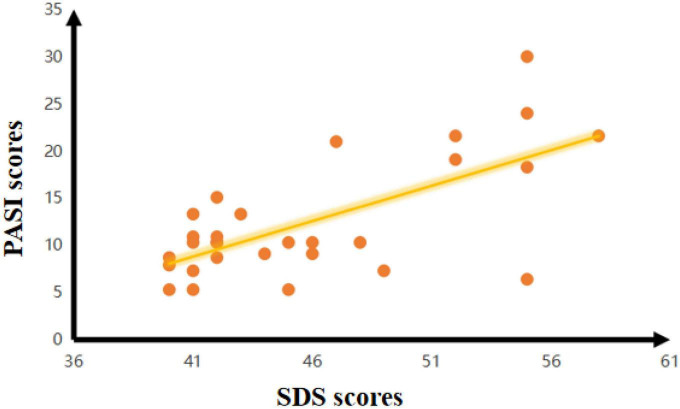
The relationships between PASI and SDS scores in PPD.

In the PPD group, there was a negative correlation between the SDS scores and FC of the hypothalamus-R and Cingulum_Mid_R (*r* = –0.414, *p* = 0.026). There was also a negative correlation between the SDS scores and zfALFF of the Frontal_Mid_L (*r* = –0.535, *p* = 0.003) in the PPD group. Moreover, PASI scores were negatively correlated with the FC of hypothalamus-R and Cingulum_Mid_R (*r* = –0.416, *p* = 0.025) and the zfALFF of the Frontal_Mid_L (*r* = –0.377, *p* = 0.044). In addition, a positive correlation between PASI and SDS scores was found (*r* = 0.509, *p* = 0.005) (Figure 3).

## Discussion

The association between psoriasis and depression is a hot spot in clinical research; however, the underlying pathogenesis of this association is still unknown. The presentation of the concept of the brain-skin axis describes correlations between brain activity and inflammatory skin disease, which provides a theoretical basis for using fMRI to explore the brain activity of PPD. To our knowledge, this study was the first to investigate the abnormal brain activity of PPD based on the theory of the brain-skin axis. In our study, compared to HCs, significantly increased fALFF values were found in the Frontal_Mid_L in psoriasis patients with depression. Meanwhile, increased FC values were found between the hypothalamus-R and Cingulum_Mid_R. Correlation analysis suggested a positive correlation between PASI and SDS scores in PPD, while the fALFF and FC values were negatively correlated with their SDS and PASI scores.

The middle frontal gyrus (MFG) is located in the frontal lobe of the cerebral cortex between the suprafrontal and subfrontal sulci and has been consistently found to be associated with processing emotional stimuli and emotional regulation (Deng et al., 2016; Peng et al., 2021). Thus, changes in the brain activity of the MFG may lead to abnormal responses to emotional events (Zhang et al., 2021). Andersson et al. (2009) showed that MFG regions are more active during cognitive control function tasks. Carter et al. (2006) showed that activity in the bilateral MFG correlates with stress and cognitive functions. Kirlic et al. (2019) reported that increased MFG activity was associated with negative affect, which is considered an important factor in the etiology of depression. Moreover, the MFG is an important part of the dorsolateral prefrontal cortex, which has been frequently found to have higher functional activity in fMRI studies of patients with depression (Kaiser et al., 2015). Abnormal brain activity in the MFG has been reported to be correlated with depression severity (Grimm et al., 2008). The MFG is a hyperactive region during depression, which is consistent with our study result showing increased fALFF values of the left MFG in PPD vs. HCs. In summary, our findings indicate that the activation of the left MFG has been consistently found to be related to social perception, the processing of social information (Völlm et al., 2006), the processing of emotional stimuli (Bermpohl et al., 2006), and emotional regulation (Ochsner and Gross, 2005). For example, Zhang et al. (2021) found significantly increased ALFF values in the left MFG in depression subjects. Zhu et al. (2022) reported a negative association between the changed FC of the MFG and the severity of depression. Che et al. (2020) reported that fALFF values of the left MFG were significantly increased in the peripartum depression group. The fALFF values of the left MFG were negatively correlated with the Hamilton depression scale (HAMD) score in patients with peripartum depression. In our study, higher fALFF values in the left MFG were negatively correlated with SDS and PASI scores in PPD. Thus, we speculated that abnormal fALFF in the left MFG plays important roles in the development of PPD.

Furthermore, the brain-skin axis provides a possible mechanism for the connection between psoriasis and depression and is mainly composed of the HPA axis. Among them, the hypothalamus is the first site for signal reception and processing of the HPA axis. In our study, psoriasis patients with depression showed increased FC between the hypothalamus-R and Cingulum_Mid_R. The middle cingulate gyrus (MCG) is located between the cingulate sulcus and the sulcus of the corpus callosum and has extensive connections with other parts of the brain through nerve fibers (Li et al., 2017; Huang et al., 2018). The MCG is regarded as a significant part of the emotional circuit and participates in processes such as regulating the body’s cognitive, emotional, memory, and self-evaluation functions (Xiao et al., 2022). Recently, an increasing number of studies have reported that the cingulate cortex is widely related to the development of depression. For example, increased anterior cingulate-inferior frontal gyrus connectivity has been reported by Rolls et al. (2019). Zhu et al. (2022) reported that the posterior cingulate showed higher resting-state functional connectivity with the right middle frontal gyrus (MFG) and the left middle temporal gyrus (MTG) in depressive patients. In addition, our correlation analysis showed that FC values were negatively correlated with patients’ SDS scores. Increasing research points to a strong relationship between cingulate gyrus FC and depression severity. For example, Smagula et al. (2021) found correlations with depression severity and amygdala-posterior cingulate FC. Ho et al. (2014) confirmed that increased anterior cingulate cortex (ACC)-amygdala FC was found in depressed adolescents and that FC was negatively correlated with depression severity. Therefore, we suggest that abnormal connectivity between the right hypothalamus and right MCG is associated with the dysfunctions of emotional processing in PPD, which could be a key point to reducing the likelihood of depression developing in psoriasis patients.

In addition, there are some reports about the relationship between the cingulate cortex and mild cognitive impairment. For example, Sambuchi et al. (2019) found gray matter atrophy in regions of the cingulate cortex in patients with mild cognitive impairment. Cera et al. (2019) showed abnormal functional connectivity of the cingulate cortex in patients with mild cognitive impairment (MCI). Furthermore, a cross-sectional evaluation showed that MCI was detected in 48.9% of psoriatic arthritis patients (Di Carlo et al., 2021). Another study conducted a systematic literature search of Embase and PubMed to identify the association between psoriasis and MCI (Yen et al., 2021). They reported that most of the 11 included studies showed a positive correlation between psoriasis and MCI. Taken together, these studies suggest that the abnormal functional connectivity of the cingulate cortex may influence the cognition of PPD. However, our study did not focus on the MCI of PPD. Therefore, the relationship between mild cognitive impairment and changes in brain activity in patients should be further studied in the future.

Our study has some limitations. Our study did not include patients with depression for comparison. Another limitation is that the sample size was relatively small. Third, the impact of the course of disease on brain FC was not considered in this study.

## Conclusion

In conclusion, our study represents a preliminary step in revealing the association between abnormal brain activity and disease severity in PPD. We demonstrated that disease severity in psoriasis patients positively correlated with their level of depression. The increases in PASI and SDS scores were negatively correlated with regional brain activity in the Frontal_Mid_L and functional connectivity between the hypothalamus-R and Cingulum_Mid_R. These findings deepen our understanding of the pathophysiological changes associated with PPD. In addition, the association between psoriasis and depression is achieved through the HPA axis-mediated immune response. Thus, future studies should combine fMRI and immune-related basic experiments to corroborate our findings.

## Data availability statement

The datasets used and/or analyzed during the current study are available from the corresponding author on reasonable request. Requests to access these datasets should be directed to XW, xxw950912@163.com.

## Ethics statement

The studies involving human participants were reviewed and approved by the Ethical Committee of the Beijing Hospital of Traditional Chinese Medicine. The patients/participants provided their written informed consent to participate in this study.

## Author contributions

GZ and XW: designing research studies. LW and NL: conduction of the study and data acquisition. NL, XW, and YZ: analyzing data. XW and NL: writing the manuscript. All authors discussed the results, commented on the manuscript, and carefully reviewed and approved the submitted version.
